# The comprehensibility continuum: a novel method for analysing comprehensibility of patient reported outcome measures

**DOI:** 10.1007/s11136-024-03858-y

**Published:** 2024-11-29

**Authors:** Victoria Gale, Philip A. Powell, Jill Carlton

**Affiliations:** School of Medicine and Population Health, University of Sheffield, Sheffield, UK

**Keywords:** Patient-reported outcomes measures, Content validity, Cognitive interview, Analysis

## Abstract

**Purpose:**

Evidence of comprehensibility is frequently required during the development of patient reported outcome measures (PROMs); the respondent’s interpretation of PROM items needs to align with intended meanings. Cognitive interviews are recommended for investigating PROM comprehensibility, yet guidance for analysis is lacking. Consequently, the quality and trustworthiness of cognitive interview data and analysis is threatened, as there is no clear procedure detailing how analysts can systematically, and consistently, identify evidence that respondent interpretations align/misalign with intended meanings.

**Methods:**

This paper presents a novel, structured approach to comprehensibility analysis - the ‘Comprehensibility Continuum’ – that builds upon existing cognitive interview guidance.

**Results:**

The Comprehensibility Continuum comprises a structured rating scale to code depth of alignment between intended item meaning and respondent interpretation and consists of five main stages: before cognitive interviews are conducted, researchers must (1) Define intended meanings of PROM items; and (2) Determine comprehensibility thresholds for both participant- and item-level. After conducting interviews, they (3) Prepare data by transcribing interviews ‘intelligent’ verbatim; (4) Code transcripts using the Comprehensibility Continuum scale in iterative sets, assigning an overall code for each item at participant-level; and (5) Compare participant-level codes across all participants to determine overall item comprehensibility, such that decisions can be made to retain, modify, or remove items.

**Conclusion:**

Quality in qualitative data analysis is achieved through rigorous methods that are clearly described and justified. Given insufficiency in guidelines, cognitive interviewers must reflect on how best to demonstrate PROM comprehensibility systematically and consistently from interview data, and the Comprehensibility Continuum method offers a potential solution.

**Supplementary Information:**

The online version contains supplementary material available at 10.1007/s11136-024-03858-y.

## Introduction

During the development and refinement of patient reported outcome measures (PROMs) used to measure health-related quality of life (HRQoL) and related constructs, evidence of acceptable content validity must be demonstrated [1–4]. PROMs should accurately reflect the construct of interest as defined in the conceptual framework [1, 2, 5] by demonstrating relevance, comprehensiveness, and comprehensibility for the intended target population [6, 7]. Without establishing content validity, PROMs may not accurately or meaningfully measure the construct they are intended to assess [2]. Conclusions about health will be inaccurate if comprehensibility is lacking i.e., respondents interpret PROM content differently to the intended meaning assumed by the researcher [6].

Cognitive interviews are widely recommended for investigating content validity and comprehensibility [2, 3, 7]. Broadly, cognitive interviews aim to understand and evaluate PROMs by collecting additional information from a participant as they complete or review the instrument to identify and address problems [8–10]. Typically, participants sampled from the PROM’s target population are asked to ‘think aloud’ (verbalise all thoughts) while completing the instrument and/or are asked direct verbal probes to gather information about their thought processes and understanding of the PROM [3]. Several thought processes and PROM components can be evaluated in cognitive interviews (Online Resource (OR): Supplement 1); here we focus on comprehension of PROM items.

Methodological guidance for analysing cognitive interview data to evaluate comprehensibility, of PROMs specifically, is lacking; the COnsensus-based Standards for the selection of health Measurement INstruments (COSMIN) suggest using an “appropriate” method of data analysis (i.e., one that is widely recognised or clearly justified) without providing further information [7]. The International Society for Pharmacoeconomics and Outcomes Research Task Force (ISPOR) state data should be used to evaluate comprehensibility and to decide whether PROM content should be retained, modified, or removed, and provide suggestions for presenting summaries of results [3]. However, exactly *how* researchers identify results, i.e., how they identify evidence for degree of comprehensibility from the cognitive interview data, is unclear. Unsurprisingly, analysis of cognitive interviews in PROM development is highly variable and reporting of analysis methods can lack detail [11].

Lack of guidance and ambiguity surrounding analysis of cognitive interviews makes it challenging for researchers to know how raw data should be used to draw conclusions about comprehensibility and content validity. Further, quality in qualitative research is ensured by researchers following rigorous, systematic processes of analysis [12, 13], that are reported in enough detail to be replicated and evaluated by others [9, 12–14]. Simply stating that data were coded for patterns from which themes (or in the case of cognitive interviewing, problems) were identified is insufficient [12–14]. Current guidance for PROM developers does not support implementation of explicit, systematic methods of comprehensibility analysis, with methods being criticised for being subjective and “impressionistic” [15] because of ambiguity and limited detailed reporting [10].

This paper describes a novel method – the Comprehensibility Continuum (CC) – that can be used in addition to existing PROM development guidance to support researchers in systematically identifying evidence for comprehensibility (or lack thereof) to enhance quality in cognitive interview analysis. The CC was developed in a project exploring the feasibility of cognitive interviewing with children aged ≤ 7 years but had broader applicability. Here we present the CC and describe the process of using the method in practice.

## The comprehensibility continuum

‘Cognitive interviewing’ is a heterogeneous methodology that can refer to several different theoretical approaches, aims, and methods of data collection and analysis [8, 9] (OR: Supplement 1). Cognitive interviewing as part of PROM development is ‘reparative’, aiming to identify if and where participants’ interpretations of items align with intended item meanings, such that problems with comprehensibility can be located and addressed [16]. Deductive coding methods of analysis in which ‘codes’ (short descriptive labels) are pre-determined at the outset of analysis and then applied ‘top-down’ to qualitative data (e.g., cognitive interview transcripts) are well-suited to this aim (for other approaches, see OR: Supplement 1). They enable researchers to pre-define what might constitute a problem with comprehensibility and provide a clear codebook to identify and label these problems consistently between analysts.

Deductive coding frameworks have been used in general survey methodology in attempts to systematise identification of questionnaire problems [17, 18], e.g., by applying “lexical” or “exclusion” problem codes [19]. However, these approaches have not clearly illustrated how researchers can identify a ‘lexical’ or ‘exclusion’ problem in the data. An operationalised approach is needed that enables (a) researchers to clearly identify what participants said that indicated where problems had occurred, and (b) consistency between researchers in making these decisions.

Christ’s continuum of young children’s semantic knowledge [20] (OR: Supplement 2) offers a potential solution in providing a framework for measuring the depth of word knowledge evident in a participant’s verbalisation, ranging from ‘0 – No Knowledge’ to ‘4 – Paired Knowledge’ [20]. For example, Level 1 word knowledge (schematically related) can be demonstrated if the participant correctly describes the emotive aspects of a word but does not fully capture the word’s meaning (OR: Supplement 2). Importantly, Christ’s continuum measures conceptual understanding of words; other methods for measuring word knowledge include, for example, phonology and syntax [21] which are not relevant to comprehensibility analysis in PROM development. The continuum was developed from a review and synthesis of pre-existing continuums [20]. Through ‘goodness of fit’ testing with over 1500 data excerpts, the operationalisation of semantic knowledge categories was confirmed, addressing limitations of previous continuums [20]. The continuum has since been applied in subsequent research (e.g [22, 23]). Although intended for young children, Christ’s continuum has potential applicability to older children and adults; it was developed from a synthesis of continuums for older children and adults, with categories of word knowledge specific to young children being incorporated *in addition* to those already synthesised from the pre-existing continuums [20]. Overall, the continuum provides a framework to support trustworthy comprehensibility analysis through the application of clear, operationalised coding.

The Comprehensibility Continuum (CC) draws upon Christ’s continuum, with several modifications made to make it applicable to cognitive interviewing in PROM development (Table 1). Technical education and linguistics language in the original continuum was removed, and health-related example responses were included. The original sub-categories ‘emerging’, ‘developing’, and ‘advanced’ (OR: Supplement 2) were intended to capture young children’s word knowledge specifically. These were removed for the CC, and sub-categories for the new levels 0–2 were kept as examples only to support applicability to a wider age range. Coding to sub-category level was also deemed too granular for the purpose of coding cognitive interviews. Additionally, the adapted CC separated ‘no/incorrect knowledge’ from ‘no relevant response’. In a cognitive interview, the analyst needs to be confident that a participant has described an item incorrectly compared to the intended item meaning as this will have implications for the potential modification of PROM content. However, no response is not necessarily indicative of no knowledge; the participant may simply choose not to answer, perhaps if they are distracted or bored.

**Table 1:** Comprehensibility continuum (CC) for coding cognitive interviews, adapted from Christ’s semantic knowledge continuum [20]

Code		Example	Definition	Example response - ‘ache’ (continuous prolonged pain in a body part)	Example response – ‘tired’ (in need of sleep or rest)
0	**No relevant response**	No response	Participant does not respond	*Silence or “errr”*,* “ummm” etc.*	
		Off-topic response	Participant responds with off-topic comment	*“I’m going to my gran’s house today”*	
1	**No/incorrect knowledge**	Incorrect meaning	Response is completely unrelated to intended meaning	*“A person who makes sandwiches”*	
		Not known	Participant says they don’t know	*“I don’t know”*,* “I can’t remember”*,* shakes head or shrugs*	
		Meaning of similar sounding word	Participant described a word that sounds similar to the target item	*“The number that comes after 7” (confuses ‘ache’ with ‘eight’)*	*“When you can’t undo your shoe laces” (confuses ‘tired’ with ‘tight’)*
2	**Schematically related knowledge** Misses the essential nature/definitive attributes of the intended meaning of the target item. The participant has partial understanding but does not clearly articulate the intended meaning either contextually (level 3) or de-contextually (level 4). There is no clear distinction between knowledge of target item and schematically related words.	Overextensions or under extensions	Participant’s description extends or restricts intended item meaning	*“It means none of your body is working” (overextension) or “it means you have bad teeth” (under extension)*	*“It means when you can’t do anything” (overextension) or “when you wake up too early” (under extension)*
		Meaning of structurally related word (i.e., target word + prefix)	Participant describes a derivation of the target item (e.g., “illness” instead of “ill”, or “well” instead of “unwell”)	*“You have a bad head” (describing a ‘headache’ rather than ‘ache’)*	*“It means that the activity is really hard” (describing ‘tiring’ rather than ‘tired’)*
		Connotation	Participant describes an idea or feeling that the target item invokes	*“It’s bad” (captures feeling/emotive knowledge associated with ‘ache’)*	*“It’s annoying” (captures a feeling associated with being tired)*
		Non-definitive attributes	Participant describes related, but not definitive, attributes related to the target item	*“You have hurt yourself” (relates to something potentially aching but does not capture essential nature of a continuous/prolonged pain)*	*“When you go to bed” (relates to potentially feeling tired but does not capture essential nature of needing sleep/rest)*
		Dummy subordinate*	Participant repeats word and uses a dummy subordinate*	*“Something aches” (‘something’ is the dummy subordinate)*	*“Somebody might feel tired” (‘somebody’ is the dummy subordinate)*
		Identified by opposite	Participant describes the opposite of the target item (e.g., “awake” instead of “sleepy”, or “happy” instead of “angry”	*“My arm is comfortable”*	*“When you feel excited and can run really fast” (describing ‘awake’)*
3	**Contextual knowledge** The intended meaning of the target item is captured in an example/meaningful context.		Response captures essential nature and must include at least one idea that is referred to by a specific noun or verb	*“Ache means when your legs are hurting all night*,* like when they are growing”*	*“Tired means when you have to go to sleep after you have stayed up past your bedtime watching a film”*
4	**De-contextual knowledge** The intended meaning of the target item is described in a way that is not couched in a contextual example.		Response shows evidence of generalisation of word’s meaning e.g., formal definition, synonym. It should not contain inaccurate information.	*“A part of your body might ache if it is painful or hurts for a long time”*	*“When you feel like you need to sleep or have a rest because you have run out of energy”*
5	**Paired knowledge**		Combines contextual and de-contextual knowledge	*“Ache means when you have something that hurts for a long time*,* like growing pains in your legs in the night”*	*“Tired is when you need to go to sleep or rest because you have used up all your energy*,* like if you watch a film really late or you spend all day playing”*

*e.g.,* “it”*,* “somebody”*,* “something”*,* “thing”*,* “people”*,* “them”*,* “stuff”.* Refers to nothing at all; it only provides a grammatical function. For example, in the sentence “It’s getting late”, the word “it” has no lexical meaning, but serves a grammatical function

### Using the comprehensibility continuum: analysis procedure

**Fig. 1:**
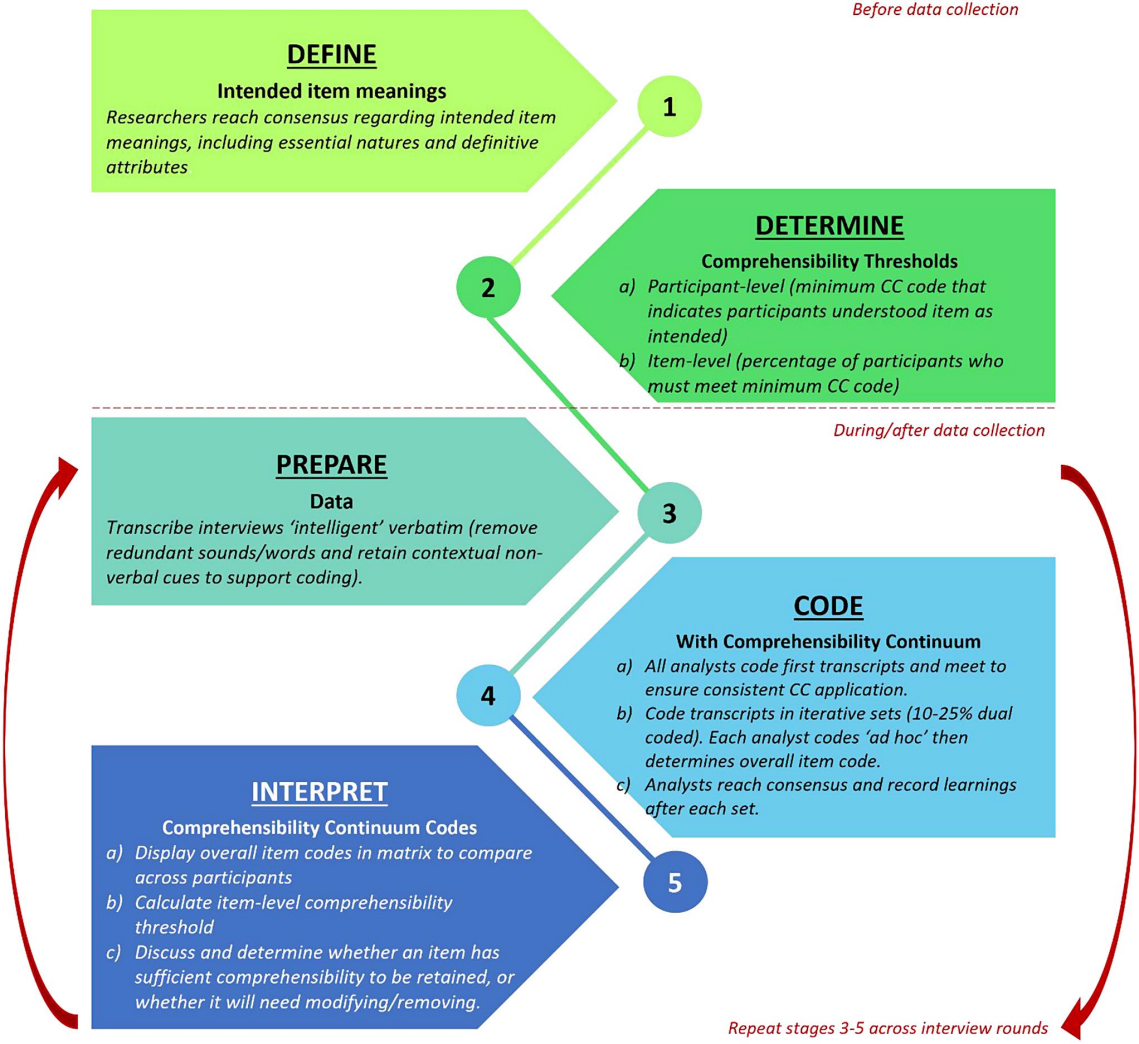
Summary of analysis process for evaluating comprehensibility using the Comprehensibility Continuum

Here we describe in detail the procedure for using the CC (Fig. 1). Illustrative examples based on research with children are used throughout.

### Define intended meanings

To evaluate overlap between intended meaning and participant interpretation, intended item meanings need defining *before* interviews are conducted. As in the original continuum [20], the CC refers to the ‘essential nature’ (i.e., fundamental meaning) and ‘definitive attributes’ (i.e., primary characteristics) of a target item, which help operationalise intended item meaning. Defining these three concepts can be applied to both simple and complex item stems; difficulty doing this may be indicative that the content of the item needs revisiting or simplifying. We recommend constructing a table that defines PROM items in this way (Table 2 – for a more detailed example, see OR: Supplement 3). Consensus must be reached between all researchers regarding the essential natures and definitive attributes of all items; the process of discussing what constitutes these definitions is particularly important for clearly describing intended item meaning. If cognitive interviews are being conducted as part of the development of a new instrument, this process will likely be informed by concept elicitation research.

**Table 2:** Example intended item meanings of items, including essential natures and definitive attributes*

Health domain	Item concept	Intended meaning	Essential nature*	Definitive attributes*
Physical	Poorly	Feeling unwell, sick, under the weather, not very well. Symptoms like headache, stomach-ache, temperature etc.	Feeling unwell/not right	Physical symptoms
	Pain	Physical hurt, a part of your body hurts	Physical hurt	A part of your body is hurting
Psychological	Sad	Feeling upset, down, low about something	Feelings of upset/down/low	Not being okay, something might have happened to make you sad. May cry.
	Angry	Feeling mad or cross about something, it might make you want to shout or cry	Feeling mad/cross about something	Physical manifestations
Social	Get on well with friends	Can join in with friends, able to talk together. Not falling out, arguing, or making each other upset – being happy with each other	Can join in with others positively	Can play and talk without arguing/falling out/making each other upset
	Lonely	Feel alone, isolated, no friends, nobody to play with or talk to	Feel alone/isolated	Nobody to talk to, play with, or be friends with

* ‘Essential nature’ refers to the fundamental intended meaning of the item. ‘Definitive attribute’ refers to the primary characteristics included within the intended meaning of the item

### Determine comprehensibility thresholds

Coding using the CC involves first coding item comprehensibility at the participant-level, and then comparing codes for each item across all participants. Before analysis begins, the research team must determine criteria for sufficient comprehensibility by agreeing thresholds at both participant- and item-level. All thresholds need to be justified and recorded a priori, such as in the study protocol.

#### Participant-level comprehensibility

Research teams will need to decide the minimum CC code necessary to indicate item comprehensibility at participant-level (i.e., that the participant is interpreting the item consistently with the intended meaning). This will be dependent on the intended target population and their unique needs. For example, Level 2 may be considered appropriate for young children (≤ 7 years) where schematically-related understanding is typical for the cognitive development of children this age [20]. Differently, if using the CC with an adult population of typical cognitive development, a minimum of Level 3 knowledge may be judged necessary. Determining participant-level comprehensibility should be tailored to each project and justified by the research team.

#### Item-level comprehensibility

When comparing comprehensibility ratings for an item across all participants it can be helpful to set a threshold for the percentage of participants who must meet the participant-level comprehensibility criteria (i.e., Level 2 for young children) for the item to be considered sufficiently comprehensible overall. We recommend ≥ 85% participants meet the minimum participant-level comprehensibility threshold. This is in-line with COSMIN guidelines for rating the sufficiency of PROM content validity, a widely used and highly regarded framework for assessing validity derived from an international consensus study [6].

### Data preparation

Current guidance recommends cognitive interviews are audio recorded and transcribed verbatim [3, 7]. To apply the CC, we recommend ‘intelligent’ verbatim transcription whereby stutters, repeated words, or sounds (e.g., “hmmm”, “ah” etc.) are retained *or* removed at the discretion of the transcriber depending on whether they are judged to be important to the meaning/context of the response. Anything relevant to the participant’s explanation of the item and that would be helpful to the analyst coding the interview should be included. For example, in the excerpt below, the participant’s hand gestures were described in square brackets to add contextual information that demonstrates the participant’s understanding of the item “walk”:

Interviewer: What does it mean if you can walk?

*Participant: [miming steps with her hands, i.e., one ‘foot’ in front of the other] “so when you can walk you can stand up and start putting your left foot first and then your right. Left*,* right*,* left*,* right. That makes you move.*

### Coding with the CC

The CC has been designed to be used to systematically code cognitive interview transcripts to support decision-making regarding item revisions across multiple interview rounds (Fig. 1). Often revisions are made to PROM items after several interviews and then re-tested in subsequent interviews, and so forth [3]. Here, we first describe the procedure for coding with multiple analysts, then we describe how to determine which CC code to apply to the data. A summary of the coding procedure is included in Fig. 1.

#### Coding procedure with multiple analysts

At least two researchers should be involved in cognitive interview analysis [7]; dual coding supports analytic quality by demonstrating consistent application of the coding frame and promotes reflexivity and discussion among the research team [24, 25]. Typically, 10–25% of transcripts are dual coded, although there is little consensus of an optimal number [24]. We suggest researchers aim to dual code a percentage within this range as is practically feasible. Analysts should agree among themselves whether the transcripts to be dual coded should be selected randomly (e.g., every fourth transcript) or whether they will be identified for specific reasons, such as the interview being challenging to conduct. Reasoning should be documented and reported. All analysts should read through their assigned transcripts for familiarisation [26–28].

Similar to framework analysis [26, 28, 29], we recommend all analysts initially code the same first few transcripts and discuss coding decisions. The aim is to reach a shared understanding of how the CC should be applied to ensure consistency in coding. All PROM items under evaluation should be included in these initial transcripts, and it can be helpful to create a table of example responses for each CC code for every item to support coding of later transcripts (OR: Supplement 3).

Transcripts should then be coded iteratively in small sets (e.g., sets of four) with analysts meeting after every set to compare and discuss coding decisions and to ensure any problems with coding are identified as soon as they arise [24]. Coding revisions made following discussion should be documented, along with the rationale for these decisions. Learnings from discussion should be documented and the analyst responsible for coding all transcripts should revise codes in non-dual coded transcripts if needed, recording all changes and reasons for these. This iterative cycle should continue until all transcripts have been coded.

#### Applying the CC to interview transcripts

In coding data against the CC, analysts select the appropriate category (Level 0–5) to characterise a participant’s response according to level of knowledge exhibited that is consistent with the intended item meaning. For example, the excerpt below was coded as ‘Level 5 – Paired’ for the item ‘sleepy’ because the participant included both de-contextual information (synonym ‘tired’) and a contextual example (feeling tired because of a lack of sleep). Further examples are included in Table 3.

*“Sleepy means when you are a bit tired. I’m a bit tired because I didn’t get any sleep today - my cat kept on waking me up.”*

Any participant response that does not capture intended meaning or is not schematically-related to intended meaning is coded as Level 0 or Level 1. This includes any explanation that may broadly be considered ‘correct’ (such as “pain” meaning *emotional* hurt) but does not capture intended meaning specific to the PROM under development/evaluation (such as if “pain” is intended to refer only to *physical* hurt). Analysts may wish to include notes or additional inductive codes alongside CC codes to capture alternative explanations of items that may be helpful for later item modifications.

With multiple analysts involved in coding, the specific ‘segments’ of transcripts coded may vary slightly across analysts [24]. We recommend analysts initially code transcripts using an ‘ad hoc data unitisation strategy’ [24] in which they determine which segments to code as they individually go through the process of coding, as opposed to formally identifying which individual ‘segments’ of transcript to code as an additional step in the analysis procedure. Substantial differentiation between analysts in identifying which section of text corresponds to which item is unlikely; cognitive interviews are typically structured by sequential consideration of each PROM item.

In some instances, the analyst will apply one code to the participant’s explanation of the item meaning. In others, they may apply multiple codes. Here, the analyst needs to determine the most appropriate overall code per participant for that item; all decisions and rationales should be clearly documented (worked examples in OR: Supplement 4). In most cases, this will be the highest code applied because typically demonstration of a lower level of understanding would not negate demonstration of a higher level of understanding. Further, if the analyst had initially included a ‘Level 3 – Contextual’ code and a ‘Level 4 – De-contextual’ code for the same item separately, these should be combined into an overall ‘Level 5 – Paired’ code for the item, as per the definition in the CC (Table 1). When analysts have determined an overall code for each item at individual transcript level, they then meet to discuss and refine codes, as described above.

However, judgement should be applied on a case-by-case basis. For example, the excerpt below initially contained both a ‘Level 4 – De-contextual’ and ‘Level 1 – No/incorrect knowledge’ code:

Interviewer: What does it mean if you’re in pain?

*Participant: “It means that you’ve hurt yourself* (4 – DE-CONTETXUAL). *Or I forgot what different kind of pain is. There’s also a different kind of pain*,* but I forgot what it is”* (1 – NO/INCORRECT KNOWLEDGE).

While overall the item was coded as Level 4 because the participant had described the intended meaning of the item (to mean physical hurt), the participant had also alluded to another interpretation of pain. This was noted alongside the overall item code for consideration when comparing the item across all participants; if other participants had also interpreted ‘pain’ to mean more than just physical hurt, this may have implications for the overall comprehensibility of this item.

**Table 3:** Example codes applied to transcripts (intended item meanings included in table 2)

Item	Participant quote	CC code	Reasoning
Poorly	*I really wanted it to land on this one*	0 – No relevant response	Off-topic response
	*[pause 8.10–8.20] I don’t know*	1 – No/incorrect knowledge	Participant says they do not know after thinking.
	*It’s not good*	2 – Schematically related	Emotional connotation associated with feeling poorly. Does not fully capture essential nature.
	*Feeling poorly is like really annoying because you might not want to cough coz someone’s talking*,* but you have to*,* and you might feel really sick that you like want to get lots of things out of you and you want to throw up.*	3 - Contextual	Participant describes several different physical symptoms that capture essential nature of intended meaning
	*It means when you are like sick and you don’t feel right and if you are a bit sick it means that you are very hot and if you feel a bit sick*,* you might need a bowl. It doesn’t feel right when you don’t feel well.*	5 - Paired	Response includes a contextual example (being sick and needing a bowl) and de-contextual, generalised explanation; it not feeling right, feeling unwell.
Get on well with friends	*When you make friends you all stay with them together. You might get lucky and stay in the same class together.*	2 – Schematically related	Participant has described non-definitive attributes of intended meaning. Staying in the same class when moving year groups may be associated with getting on well with one another but does not capture essential nature.
	*It means that you don’t just say ‘no I don’t want to play with you’. You play with them*,* and they don’t say that you’re mean or angry or really mean they just get along with you.*	5 - Paired	Specific examples of what ‘getting on well’ with a friend would involve (not being mean or angry, letting each other play) that capture essential nature, and synonym “get along with” suggesting generalised knowledge.
Angry	*It means when you maybe feel like to punch a little bit*	3 - Contextual	Specific example that captures essential nature of intended meaning
	*Angry means when you’re mad.*	4 – De-contextual	Generalised knowledge shown through a synonym that captures essential nature of intended meaning.
	*It means that you’re out of conscience and you make a big mess.*	5 - Paired	Response includes a contextual example that captures essential nature (making a mess) and a generalised de-contextual description of acting out of conscience when angry.

### Interpretation of CC codes

Current guidance advises that cognitive interview data is used to make decisions to retain, modify, or remove items based on participant comprehensibility [3]. The CC codes applied to transcripts must therefore be interpreted to inform this decision-making process. Overall comprehensibility ratings for each participant should be displayed in a matrix to support comparison at item-level (Table 4). From this, the percentage of participants who meet the minimum CC code threshold (≥ Level 2 in Table 4) can be calculated for each item. As shown in Table 4, the item-level threshold of ≥ 85% participants reaching ≥ Level 2 was met for all three items, indicating the items have sufficient comprehensibility and can be retained in their current form. However, equal importance should be given to the discussion between analysts when determining whether an item should be retained, modified, or removed; the minimum percentage threshold can act as a helpful guide, but is fundamentally arbitrary and should be used in conjunction with knowledge of interview context and analysts’ reflections.

### Reporting cognitive interview results

Results of analysis with the CC should be reported in line with recommendations [3]. This can include an item tracking matrix to show the development of each item over the course of the cognitive interviews, including the rationale behind decisions to keep, modify, or remove items. Participant quotes illustrating the comprehensibility of each item should also be included i.e., example quotes for the different CC codes applied to each item. Reporting detail is highly variable across cognitive interview studies conducted as part of PROM development [11] and we recommend increased transparency overall.

**Table 4:**
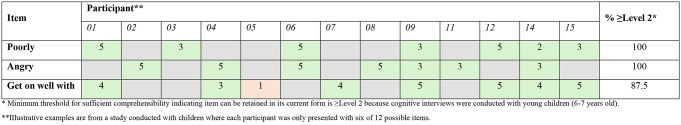
Example comparison of overall comprehensibility ratings across participants

## Discussion

Here we have outlined an approach for systematically analysing cognitive interview data that can be used by researchers in addition to existing guidelines. The Comprehensibility Continuum can be applied to code data and summarise the extent of overlap between participants’ interpretations of, and intended meanings of, PROM content. While there is no single correct way to analyse cognitive interview data [16], current guidelines do not sufficiently detail how researchers can evaluate PROM comprehensibility, thus undermining assessments of content validity during instrument development. The CC addresses this unmet need for a systematic approach to analysis.

The CC method draws on the strengths of pre-existing approaches to qualitative data analysis to address the main limitations in current cognitive interview analysis guidelines. It uses an adapted version of Christ’s continuum of semantic knowledge [20] to operationalise PROM comprehensibility. This enables analysts to systematically identify evidence for mis/alignment between participant interpretation and intended meaning of PROM content by providing tangible parameters for what constitutes alignment. Such guidance is missing from current recommendations by ISPOR [3] and COSMIN [7]. Despite being based on a continuum developed for young children, the CC has potential to be applicable to any age group; the original continuum was developed from a synthesis of continuums for both children and adults [20]. Furthermore, while we have presented examples from research conducted with children, researchers may choose to adjust thresholds according to participant age and the principles of operationalising mis/alignment between intended meaning and participant interpretation are transferable across age groups. Deductive coding strategies are not new in cognitive interview analysis [16], but to our knowledge none have operationalised comprehensibility in this way. Using the CC allows analysts to clearly describe how and why codes were applied to interview data, necessary for ensuring trustworthiness and quality [12, 13].

The CC method is grounded in the cognitive theory that underpins cognitive interviewing (i.e., that respondents’ cognitive processes underlie survey responses and that these can be made visible through interview methods [8, 30]) and the reparative aims of cognitive interviewing that are typical of this research as part of PROM development. Researchers can use the CC codes to evaluate comprehensibility across all participants and use this to then inform decisions regarding PROM modification, as recommended by ISPOR [3]. It offers a systematic, replicable, and theoretically grounded approach to cognitive interview analysis.

It is important to emphasise that the CC is intended for use in cognitive interviewing as part of the development/refinement processes of PROMs where evidence for comprehensibility for a specified context of use and target population is needed [1–6]. This is different from the PROM application context (e.g., data collection in clinic) where evaluations of validity should focus on the extent instrument scores reflect the individual respondent’s health/HRQoL [31, 32]. Cognitive interviewing cannot identify *all* possible comprehensibility problems that might arise in PROM application [8]. Instead, it is a recognised practical solution for improving instrument design and providing the evidence for comprehensibility required as part of PROM development [1–6]. The CC can support identification of this evidence.

In proposing a deductive coding scheme with a structured approach to implementation and dual coding we do not suggest the more interpretivist, descriptive approaches to cognitive interview analysis are lacking in rigour or quality. Nor do we suggest that cognitive interview analysis is an objective endeavour. Rather, we aim to emphasise the need for cognitive interviewers to critically reflect on what constitutes evidence of PROM comprehensibility and use transparent methods for evaluating this. Achieving high inter-rater reliability scores to ‘tick a box’ on a research checklist is not the purpose of the CC; instead, discussion and achieving consensus amongst researchers is where reflective insights about the data are learned and where analysts will develop a systematic approach to coding [12].

The CC has potential to support the design of cognitive interview studies. ISPOR provide recommendations for verbal probes to evaluate comprehensibility [3] and the CC may also influence probes asked; for example, interviewers may specifically probe for contextual examples or generalised definitions of the target item. The CC approach is not without limitations. While the thresholds described are informed by pre-existing literature and could be a guide to others, they are fundamentally arbitrary. Researchers must discuss and agree amongst themselves what would constitute sufficient evidence of item comprehensibility at participant-level, and how many participants must meet this threshold to indicate that an item can be retained without modification. The CC is intended to support best practice standards set, for example, by ISPOR [3] and COSMIN [6] whereby items should be understood as intended for use. There may be instances where the need to establish uniformity of comprehensibility across a target population is less important (e.g., PROMs developed solely to support clinical observations). Here developers may not wish to evaluate uniformity of item-level comprehensibility, but the CC can still support in initial development processes to evaluate understandability of items, as required.

The CC only addresses evaluation of item comprehensibility. Overall relevance and comprehensiveness are also important factors to consider when evaluating content validity [6] and so should also be addressed by researchers. However, the underlying principle of critical reflection regarding what constitutes relevance and comprehensiveness should be transferred. Similarly, comprehensibility problems can arise in other PROM components, such as completion instructions and response options. While the CC was developed in a project specifically focussed on evaluating item comprehensibility, again the principle of critical reflection regarding what constitutes comprehensibility could be transferred to other PROM components (e.g., instructions, response options, recall period); further consideration of the applicability of the CC to these components has yet to be explored. The CC was initially applied successfully in a cognitive interviewing study with young children, but independent validation by research teams will be needed.

## Conclusion

Establishing content validity of PROMs is essential to ensure accurate measurement of HRQoL and related constructs. Cognitive interviews are widely recommended and used to achieve this, yet analysis procedures are insufficiently described in cognitive interview guidelines. Here we have detailed the CC, an approach to analysis that is applicable to any participant age-group. The CC method allows for systematic assessment of comprehensibility and will serve as an addition to current guidance for researchers providing supportive evidence for content validity.

## Electronic supplementary material

Below is the link to the electronic supplementary material.

Supplementary Material 1
