# Supplementary material for: The comprehensibility continuum: a novel method for analysing comprehensibility of patient reported outcome measures
Source: Qual Life Res. 2024 Nov 29;34(4):937–47. doi: 10.1007/s11136-024-03858-y (PMC11982068; doi:10.1007/s11136-024-03858-y)
Supplement: Supplementary file 1 — Supplementary Material 1 [file 11136_2024_3858_MOESM1_ESM.pdf]

Online Resource

Article Title: The Comprehensibility Continuum: A Novel Method for Analysing Comprehensibility of Patient Reported Outcome Measures

Journal: Quality of Life Research

Authors: Victoria Gale[a], Philip Powell[a], and Jill Carlton[a]

Affiliation: [a]School of Medicine and Population Health, University of Sheffield, UK

Corresponding author: Victoria Gale; vargale1@sheffield.ac.uk

Table of Contents

Supplement 1 - Background to Cognitive Interviewing.......... 2

Supplement 2 – Continuum of Young Children's Semantic Knowledge.......... 5

Supplement 3 – Example intended item meaning and example anticipated responses.......... 7

Supplement 4 – Worked examples for determining overall item codes.......... 8

Supplement 1 - Background to Cognitive Interviewing.

A short overview of the origins, theory, and different aims and analysis methods of cognitive interviewing in general survey methodology and in the development of patient-reported outcome measures (PROMs).

Cognitive interviewing as originally conceptualised was based on cognitive theory; cognitive processes were theorised to underpin responses to survey questions, with Tourangeau's four-stage model [1] being particularly influential (Figure 1) [2-4]. Methods of think aloud and verbal probing in a cognitive interview were thus theorised to make these cognitive processes visible such that the survey could be evaluated for response error [2-4]. Cognitive theory continues to underpin cognitive interviews as applied to PROM development today, as can be seen with the emphasis on needing to understand and evaluate how respondents comprehend PROM content, recall relevant information, and create a response [5].

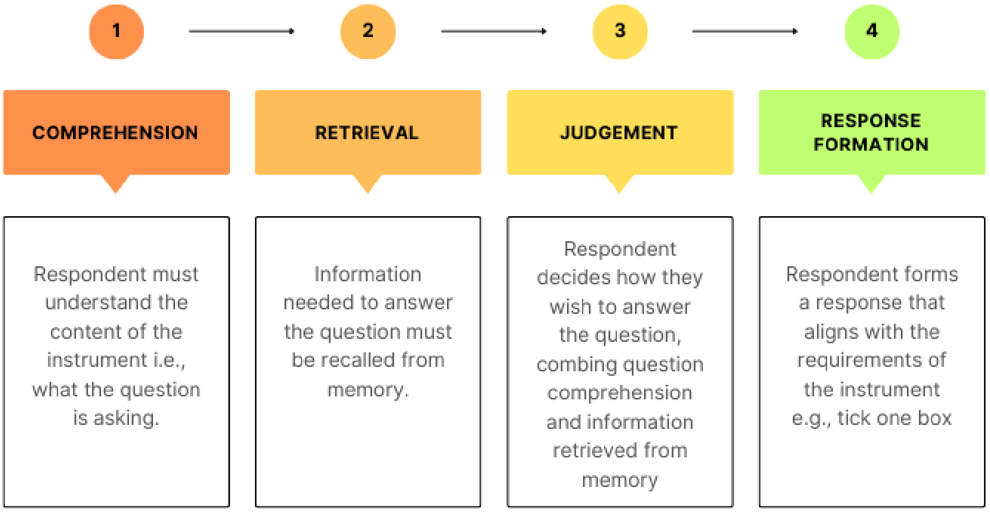

Fig. 1 Tournageau's four-stage model of cognitive processing theorised to underpin survey responses [1].

However, it was increasingly recognised that cognitive processes alone do not drive survey responses [3-4]. For example, motivation is a key contributor in determining how an individual responds to a questionnaire [2]. Similarly, there can be many 'non-cognitive' benefits to conducting a cognitive interview, such as identification of problems with relevance and comprehensiveness, or practical problems with the formatting and layout of the instrument [4]. Again, this can be seen in cognitive interviews as applied to PROM development. Interpretivist cognitive interviews also adopt "cognitive plus" theories; an individual's survey response is not thought to occur in a cognitive vacuum, but rather their thought processes interact with their lived experiences and social environment [6-7].

Cognitive interviewing aims typically fall along a continuum from Reparative to Descriptive [4, 8, 9]. Reparative interviews aim to identify and repair problems with the instrument while Descriptive interviews aim to describe the meaning of a survey statistic by identifying and explaining the phenomena captured by the survey question [4, 8, 9]. Cognitive interviews for PROM development have largely Reparative aims; ISPOR and the FDA recommend cognitive interviews are used to identify problems with respondent comprehension and with content comprehensiveness, and PROMs should be modified as a result of interview findings [5, 10]. Differently, interpretivist cognitive interviews do not assume that there is a correct interpretation of survey questions [6, 7]. Instead they have a Descriptive aim that intends to identify and explain the overall functioning of a survey question in an effort to understand the meaning of the data gathered from a survey [6, 7].

Three overarching approaches to cognitive interview analysis reportedly exist more generally: (1) Uncoded text summaries; (2) Inductive coding; and (3) Deductive coding (Table 1) [8, 9]. Inductive coding approaches are more typically aligned with interpretivist and descriptive approaches to cognitive interviewing [6, 7].

Table 1. Approaches to cognitive interview analysis identified within general survey methodology [8]

**Overarching approach**	**Sub-category**	**Definition**
**Uncoded – Text Summary**		Interviews are summarised in a written description of the major themes and problems identified with the instrument.
**Inductive Coding**<br>*Codes identified 'bottom-up' from the data*	Theme Coding	Themes are developed from codes that capture the overall functioning of the content of the instrument (i.e., a thematic analysis).
	Pattern Coding	Researcher searches for patterns that illustrate how the functioning of the instrument content varies as a function of other factors (e.g., respondent's socio-economic context).
**Deductive Coding**<br>*Codes pre-determined and assigned 'top-down' to the data*	Cognitive Coding	Codes are applied that illustrate cognitive processes e.g., based on Tourangeau's four-stage model [1].
	Question-feature Coding	Codes applied that capture question features e.g., ambiguous language, complex terminology, unspecified recall period etc.

References:

1 Tourangeau, R. (1984). Cognitive science and survey methods: a cognitive perspective. In Jabine, T., Straf, M., Tanur, J., Tourangeau, R. (Eds.). *Cognitive Aspects of Survey Methodology: building a bridge between disciplines.* National Academy Press.

2 Beatty, P.C., & Willis, G.B. (2007). Research Synthesis: The practice of cognitive interviewing. *Public Opinion Quarterly, 71*(2), 287-311. https://doi.org/10.1093/poq/nfm006

3 Boeije, H., & Willis, G.B. (2013). The Cognitive Interviewing Reporting Framework (CIRF). *Methodology, 9*(3), 87-95. https://doi.org/10.1027/1614-2241/a000075

4 Willis, G.B. (2005). *Cognitive interviewing: a tool for improving questionnaire design*. Thousand Oaks.

5 Patrick, D.L., Burke, L.B., Gwaltney, C.J., Leidy, N.K., Martin, M.L., Molsen, E., & Ring, L. (2011). Content Validity—Establishing and Reporting the Evidence in Newly Developed Patient-Reported Outcomes (PRO) Instruments for Medical Product Evaluation: ISPOR PRO Good Research Practices Task Force Report: Part 2—Assessing Respondent Understanding. *Value in Health, 14*(8), 978-988. https://doi.org/10.1016/j.jval.2011.06.013

6 Miller, K., Chepp, V., Wilson, S., & Padilla, JL. (2014). *Cognitive Interviewing Methodology.* Wiley & Sons

7 Miller, K. (2011). Cognitive Interviewing. In Madans, J., Miller, K., Maitland, A., & Willis, G. (Eds.). *Question Evaluation Methods: Contributing to the science of data quality*. John Wiley & Sons.

8 Willis, G.B. (2015). *Analysis of the cognitive interview in questionnaire design.* Oxford University Press.

9 Meadows, K. (2021). Cognitive interviewing methodologies. *Clinical nursing research, 30*(4), 375-379.

Supplement 2 – Continuum of Young Children's Semantic Knowledge

Christ's [1] continuum of young children's semantic knowledge. This was then adapted for use in cognitive interview analysis – the Comprehensibility Continuum as discussed in the main text.

Level		Category	Definition
**0**	**No knowledge**	No response	Child says they do not know the word's meaning
		Incorrect meaning	Response is completely unrelated to the target word (or a similar sounding word)
		Meaning of a phonologically similar word	Child confuses the target word with a word that is similar sounding but unrelated in meaning
**1**	**Schematically related knowledge**	Overextensions and under-extensions	Meaning of the word is extended or restricted beyond its actual limits
		Meaning of morphologically related word	Correct description of a derivation of the target word (e.g., 'rescuer' instead of 'rescue')
		Connotation	Correct demonstration of emotive understanding but no more specific knowledge of word's meaning
		Non-definitive attributes	Description of a related attribute to the word, but does not capture essential nature
		Syntactic placement with dummy subordinate	Word is briefly used in an example with a dummy subordinate e.g., 'stuff', 'thing', or 'somebody'
		Identified by opposite	Correct explanation of a word opposite to the target word
**2**	**Contextual knowledge**	Emerging	Uses at least one idea referred to by a specific noun or verb that captures the word's essential nature in a contextual example
		Developing	
		Advanced	
**3**	**De-contextual knowledge**	Emerging	Description of the word's meaning using a synonym, that captures the essential nature, definitive attributes, or a superordinate. Cannot be couched in a contextual example and must not include inaccurate information
		Developing	
		Advanced	
**4**	**Paired knowledge**	Emerging	Child articulates both contextual and de-contextual knowledge

References:

1 Christ, T. (2011). Moving past “right” or “wrong” toward a continuum of young children’s semantic knowledge. *Journal of Literacy Research, 43*(2), 130-158. https://doi.org/10.1177/1086296X11403267

Supplement 3 – Example intended item meaning and example anticipated responses

Here we provide an example of a more detailed intended item meaning ("play") and provide example content anticipated to be included in participant responses for levels 2-5 of the Comprehensibility Continuum using the target item "lonely".

Example intended meaning for the item concept "play". Please note that this intended meaning is specific for play by infants and toddlers (0-3 years).

Item concept	Intended meaning	Essential nature	Definitive attributes
**Play**	This includes engagement with fine motor and gross motor activities for exploration and can include mouthing, swiping/manipulation of objects or toys (e.g., mobiles, clothing, blankets, toys, household items or items within their environment e.g. stones, leaves, mud, water). This will develop into fetching, carrying, posting, passing, throwing, banging, pushing, kicking, jumping, cycling, creation of music, dancing or art etc. This can include structured play (where play is set up and guided) or unstructured play. It can be child led or led by someone else. Toddlers may begin imaginary play without items. Older toddlers may play alongside each other in similar activities.<br><br>*To note this is not limited to interaction with commercially available items such as toys. Many will consider this fun*	Exploration of or engagement with objects, sounds, body, imagination. Differs from movement as it does not necessarily have to be goal directed or rewarded. Together or alongside of others. For enjoyment, fun and or learning.	Enjoyable engagement with objects, sounds, body and/or imagination.

Example content anticipated to be included in participant responses

Code	CC Level	Example content for "lonely"
**2**	Schematically related knowledge	You have to tell a teacher (non-definitive attribute)<br>It's boring (connotation)<br>It means there is just one (described 'only')<br>You have loads of friends (opposite)<br>Somebody feels lonely (dummy subordinate)
**3**	Contextual knowledge	You don't have any friends to play with at lunch time or play time.
**4**	De-contextual knowledge	You don't have any friends, you have nobody to talk to or play with – you are alone.
**5**	Paired knowledge	You are alone with no friends. At lunch time you have to play by yourself

Supplement 4 – Worked examples for determining overall item codes

Worked examples for determining the overall Comprehensibility Continuum rating for each item at participant-level.

Example 1:

Overall rating is highest level coded - 'Level 3 – Contextual'. The segment coded at Level 2 does not negate or contradict the segments coded at Level 3.

Interviewer:	Can you tell me what it means if somebody does feel poorly?	
Participant:	*If someone does feel poorly it means that they might have to go to the hospital or if it's not that bad they can go to school*	**2 – Schematically related.** Non-definitive attributes (going to school or hospital)
Interviewer:	Absolutely, and can you tell the teddy bear what feeling poorly is like?	
Participant:	*Feeling poorly is really annoying because you might not want to cough coz somebody's talking, but you have to and you might feel really sick that you like want to get lots of things out of you and you want to throw up*	**3 – Contextual.** Examples of physical symptoms (coughing, feeling sick) that capture essential nature.
Interviewer:	I know what you mean, so feeling poorly, it can be like coughing or feeling sick? Can it be anything else?	
Participant:	*It could be like you're too poorly so like you're too tired and you don't have any energy so you might have to stay home and not go to anywhere that you're supposed to go*	**3 – Contextual.** Examples of physical symptoms (tired, lacking energy) and definitive attributes.

Example 2:

Overall rating is 'Level 5 – Paired' because the Comprehensibility Continuum defines Level 5 as when the participant includes both Levels 3 and 4 in their explanation.

Interviewer:	So what does it mean if you're really scared?	
Participant:	*In case I get told off by my teacher and I don't like being told off*	**3 – Contextual.** Examples of situations that would make someone scared that capture intended meaning.
Interviewer:	No	
Participant:	*And in case I do something wrong.*	
Interviewer:	That is definitely something that can make us feel scared I know what you mean – can you explain to the teddy bear what being scared feels like?	
Participant:	*So it always happens to us these feelings and feeling scared means like you're afraid of something or you're afraid if something's going to happen*	**4 – De-contextual.** Generalised description of feeling scared capturing essential nature and using synonym 'afraid'.

Example 3:

Overall rating is 'Level 5 – Paired' because the participant's responses include both a Level 3 and Level 4 explanation. The Level 1 verbalisation does not contradict the Level 3 and 4 explanations and was judged by the analyst to be a problem with the cognitive interview probe; this participant was 6 years old and thinking of synonyms can be a cognitively challenging task for this age group.

Participant:	*It's because I'm a bit lonely because I've not really talked to a lot of people, I've just mainly run around and I feel just a bit lonely*	**3 – Contextual.** Specific example of why they feel lonely that captures essential nature of intended meaning.
Interviewer:	So can you tell me a bit more about what lonely means?	
Participant:	*Lonely means when you don't feel like someone's near you, like very far away from you and that someone doesn't like you*	**4 – De-contextual.** Generalised description of feeling lonely capturing intended meaning.
Interviewer:	Can any other words mean lonely?	
Participant:	*I don't quite know really*	**1 – No/incorrect knowledge.**
